# Preparation and Properties of Natural Bamboo Fiber-Reinforced Recycled Aggregate Concrete

**DOI:** 10.3390/ma17122972

**Published:** 2024-06-18

**Authors:** Binyu Xu, Rongxi Tian, Ying Wang, Zhen-wen Zhang, Zihua Zhang

**Affiliations:** School of Civil & Environmental Engineering and Geography Science, Ningbo University, Ningbo 315211, China

**Keywords:** natural bamboo fiber, recycled concrete, orthogonal experiment, microstructure

## Abstract

To promote resource reuse and the green, low-carbon transformation of the construction industry, this study uses recycled aggregate from crushed waste concrete and natural bamboo fibers to formulate bamboo fiber-reinforced recycled-aggregate concrete. This study investigates the effects of natural bamboo fiber (NBF) content, NBF length, and the water-to-cement ratio on the performance of concrete through an orthogonal experiment to determine the optimal mixing proportions of NBF-reinforced concrete. Additionally, recycled aggregate completely replaced natural aggregate. The mechanism by which NBF influences concrete was also analyzed. The results demonstrate that the NBF-reinforced specimens exhibited good integrity during compression failure, with NBFs effectively tying the concrete together. The optimized parameters for NBF-reinforced concrete were an NBF length of 20 mm, an NBF content of 0.4v%, and a water-to-cement ratio of 0.55. Almost no flaky Ca(OH)_2_ crystals were observed in the NBF-hardened cement–paste transition zone, indicating effective bonding at the interface.

## 1. Introduction

Urbanization results in the depletion of significant natural resources and the production of construction waste due to the erection of new buildings and the dismantling of old ones [1]. Typically, waste concrete is disposed of via landfilling and stacking, resulting in the substantial wastage of land resources [2,3]. The reuse of recycled aggregate obtained from crushed, sieved, and cleaned waste concrete in concrete structures stands as a crucial method for the utilization of construction waste resources [4,5,6,7].

The surface of recycled aggregate is coated with a certain amount of mortar, resulting in increased porosity and water absorption compared to natural aggregate. Furthermore, recycled aggregate often contains numerous micro-cracks, leading to inferior properties compared to conventional concrete [8,9,10,11]. Incorporating fibers into recycled concrete can partially offset the deficiencies of recycled concrete by enhancing toughness and crack resistance [12,13]. Typical fibers used in concrete include steel, polypropylene, glass, carbon, basalt, and natural fibers [14].

Steel fibers are effective in impeding crack propagation, while polypropylene fibers are cost-effective and long-lasting; therefore, they are the most commonly utilized types in recycled aggregate concrete [15,16]. In their study, He et al. [17] combined polypropylene and steel fibers in recycled aggregate concrete and found that the combined impact of both fibers on recycled aggregate concrete mechanical properties surpassed that of either fiber alone. Afroughsabet et al. [18] incorporated 1% double hooked-end steel fibers into recycled aggregate concrete and observed significant enhancements in tensile strength, alongside reductions in shrinkage and water absorption. Zheng et al. [19] improved recycled aggregate concrete with nano-SiO_2_ and basalt fibers, resulting in a 34.28% increase in 28-day compressive strength, a 40.55% increase in splitting tensile strength, and a 54.5% increase in flexural strength post-modification. Carneiro [20] demonstrated that incorporating steel fibers boosted the mechanical strength and toughness of recycled concrete.

Although the utilization of steel and polypropylene fibers in recycled concrete has been extensively researched, there is a growing need to explore the more efficient integration of high-performance natural fibers with a low carbon footprint and biodegradability. Prior studies have investigated the use of natural fibers like hemp, kenaf, and bamboo in concrete, demonstrating the feasibility of plant fibers in enhancing concrete performance [21]. Bamboo resources, which are abundant globally, have a short growth cycle and require little energy for harvesting and processing, providing energy-saving advantages. Bamboo has a cellulose content of 40–60%, hemicellulose content of about 20%, and lignin content of about 25%. The mechanical characteristics of bamboo fluctuate based on species, age, and geographical origin. Typically, bamboo exhibits a moisture content (in an air-dry state) of approximately 8%, a dry shrinkage rate ranging from 9% to 15%, a density between 0.8 and 1.2 g/cm^3^, a compressive strength of about 90 MPa, a bending strength of about 250 MPa, and a tensile strength of about 300 MPa [22]. Nie et al. [23] conducted experimental and analytical examinations of the axial compressive characteristics of recycled aggregate concrete columns confined by bamboo tubes. Their findings revealed that the incorporation of short bamboo strips could enhance the properties of recycled concrete columns. The bamboo tube structure offered robust lateral confinement, while the short bamboo strips augmented both the compressive strength and ductility of recycled aggregate concrete columns. In a similar vein, Noh et al. [24] employed bamboo fibers to substitute aggregate and compared the compressive strength of bamboo fiber-reinforced concrete with that of standard concrete. They determined an optimal bamboo fiber content of 5% for aggregate replacement in concrete.

Bamboo fiber can be categorized into charcoal bamboo fiber, pulp bamboo fiber, and natural bamboo fiber. Pulp bamboo fiber is produced through the pulping of bamboo material, whereas charcoal bamboo fiber is derived from bamboo that has been carbonized into micro-powder and bonded with viscose material. Both processes completely destroy the structure of the bamboo material and are not environmentally friendly. Natural bamboo fibers (NBFs) are directly separated from bamboo. They are elongated in shape, with stiff fibers, rough surfaces, and shallow grooves. The cross-section is nearly round with irregular jagged edges. They retain the natural physical structure of virgin bamboo and have a high specific modulus and high specific strength, making them high-performance renewable fibers [25]. Additionally, using bamboo fibers in building materials has excellent environmental characteristics, contributing to carbon emission targets since plant fibers can store carbon.

However, natural fibers exhibit certain limitations in concrete applications, such as insufficient bonding at the fiber–concrete interface, low hydrophilicity, and vulnerability to biodegradation. Concurrently, several studies have demonstrated that alkali treatment can enhance the bonding strength between fibers and the cement matrix, as well as improve hydrophilicity [26,27,28]. Wei et al. [29] explored the durability of sisal fiber-reinforced cementitious materials under wet and dry cycling, revealing an 86% decrease in the tensile strength of fibers embedded in the cement matrix after 30 cycles. Wang et al. [30] discovered that flax fiber-reinforced polymer (FFRP) reduced flexural strength by 11.2%, 14.9%, and 15.5% and flexural modulus by 21.3%, 32.3%, and 35.8% after natural aging for 60, 120, and 180 days, respectively. Chakkour et al. [31] demonstrated that exposing plant fiber composites to a wet environment significantly diminishes their mechanical properties.

Additionally, Chakkour et al. [32] experimentally determined that the tensile strength and Young’s modulus of bamboo fiber composites decreased by 18.15 ± 0.34% and 18.55 ± 0.4%, respectively, after 120 days of immersion. Akinyem et al. [33] assessed the impact of 50 cycles of wet/dry processes on plant fiber cementitious composites, noting a slight increase in pore size before and after aging tests, with improved dimensional stability when sodium hydroxide was used at concentrations between 0 and 2.0%. Geremew et al. [34] investigated the effect of alkali treatment on the surface morphology of bamboo fibers. Their findings strongly suggest that bamboo fibers prepared using these techniques could be employed as reinforcing material in composite production. Durability tests indicated that immersing bamboo culm in water for a month enhances its serviceability. This method could reduce reliance on harmful chemical preservatives [35].

However, existing studies on the properties of NBF-reinforced concrete are limited, underscoring the necessity for further investigation. While many studies have delved into the impact of fiber admixture and length on concrete, it is imperative to broaden the scope of inquiry to encompass the water–cement ratio, given the substantial influence of bamboo fibers’ high water absorption on concrete workability and mechanical properties.

This study investigates the impact of the water–cement ratio, fiber admixture, and fiber length on the overall performance of NBF-reinforced concrete. It examines how NBF content, NBF length, and the water-to-cement ratio affect the workability, compressive strength, splitting tensile strength, and flexural strength of concrete through orthogonal experimentation, with the goal of determining the optimal NBF proportion. Additionally, all coarse aggregates were replaced with recycled aggregate, and the influence mechanism of NBFs on concrete properties was analyzed using scanning electron microscopy (SEM). The study utilized the orthogonal experiment design method, a scientific approach for investigating multifactorial and multilevel experiments. This method selects representative points from a full-scale experiment based on orthogonality, allowing for the comprehensive analysis of various factors.

The results of this study provide a basis for furthering the practical application of NBF-reinforced recycled concrete. They also serve as a reference for innovative approaches to solid waste reuse and the development and adoption of low-carbon biomass fibers. Ultimately, this can accelerate the utilization of environmentally friendly, low-carbon building materials.

## 2. Overview of the Experiment

### 2.1. Materials and Pretreatment

The raw materials comprised PO42.5 Portland cement, Grade II fly ash, recycled aggregate sourced as the coarse aggregate (obtained from a recycled resource utilization company in Ningbo, Zhejiang Province, China), desalinated sea sand serving as the fine aggregate, and tap water.

The NBFs used in this study were produced from Moso bamboo by a company in Sichuan Province. The NBFs were natural fibers directly separated from bamboo through a combination of mechanical and physical filament splitting, biological degumming, and opening and carding. Impurities were removed through refining, flaking, crushing and decomposition, bio-enzymatic degumming, fiber carding, and sieving. The entire NBF production process was conducted under slightly alkaline conditions. The bamboo fibers were decomposed by bio-enzymes, resulting in a finished product with good alkali resistance without alkaline cooking treatment.

**Figure 1:**
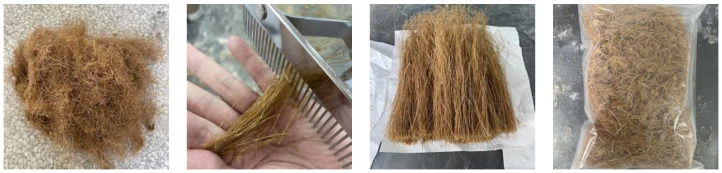
Carding and cutting of NBFs.

**Figure 2:**
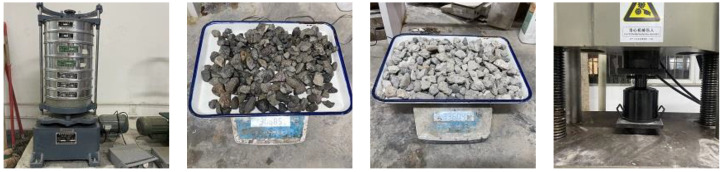
Testing of the material properties of recycled aggregate.

Before concrete casting, as shown in Figure 1, the NBFs were pretreated by carding the fibers and cutting them into different lengths for use. The particle gradation, apparent density, water absorption, and crushing index of the recycled aggregate were evaluated to assess its quality grade, as depicted in Figure 2.

A 7 kg sample of recycled aggregate was extracted for a sieving test, then dried until it achieved a constant weight, and subsequently sieved in batches. The square-hole vibrating sieve had apertures ranging from 2.36 mm to 31.5 mm, arranged in ascending order, with a cover placed on top for the sieving process. After the sieving process was completed, the residue remaining on each sieve was weighed and calculated to determine both the percentage of the subtotal and the cumulative percentage for each sieve aperture.

**Figure 3:**
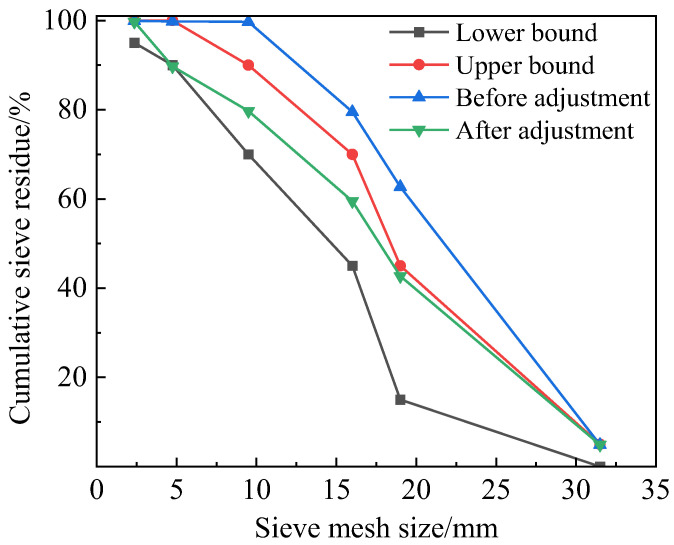
Gradation curve of recycled aggregate.

The sieving results of the aggregate are shown in Table 1. Cumulative screen residue refers to the proportion of aggregate that fails to pass a given sieve size. The directly recycled aggregate had a relatively large overall particle size, and therefore it was imperative to modify the gradation by decreasing the proportion of coarse aggregate with particle sizes ranging from 19 to 26.5 mm to 20% and adjusting the proportions of coarse aggregate with particle sizes ranging from 4.75 to 9.5 mm and 2.36 to 4.75 mm to 10%. This particle size distribution met the gradation requirements for the preparation of concrete, as shown in Figure 3. The measured values of various parameters are shown in Table 2.

### 2.2. Mix Design and Sample Preparation

The mixing ratios of C30 NBF-reinforced concrete were primarily established following the guidelines outlined in the Specification or Mix Proportion Design of Ordinary Concrete (JGJ 55-2011) [36]. Following the preliminary design, trial mixing, and subsequent adjustments, the final mixture of C30 NBF-reinforced concrete with varying water–cement ratios for this experiment was determined, as detailed in Table 3. The fly ash content constituted 20% of the cementitious material.

**Figure 4:**
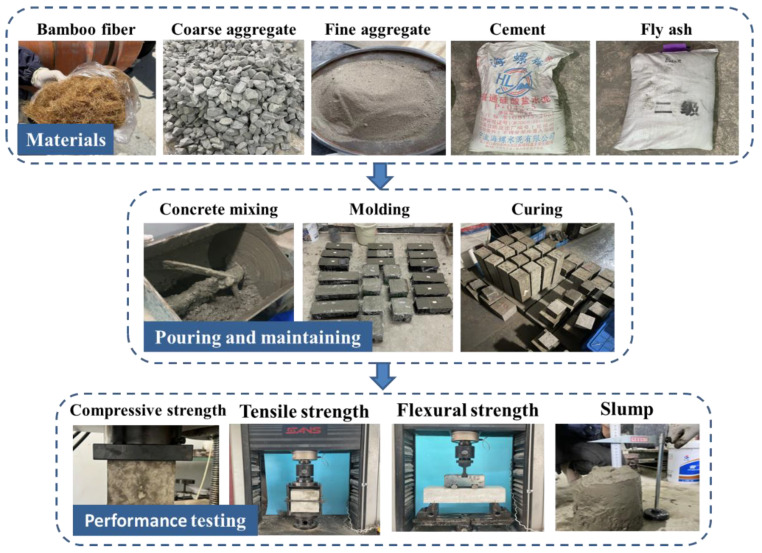
Casting and property testing of NBF-reinforced concrete specimens.

The concrete was poured following the guidelines outlined in the Standard for Test Methods of Concrete Physical and Mechanical Properties (GB/T 50081-2019) [37]. Specimens for compressive strength and splitting tensile strength tests were sized at 150 mm × 150 mm × 150 mm, while specimens for flexural strength tests measured 100 mm × 100 mm × 400 mm. The poured specimens underwent natural curing in molds for one day. Subsequently, they were maintained under standard conditions with a temperature of 20 ± 2 °C and a humidity level of ≥95%, where they were cured for 28 days. Figure 4 illustrates the casting and property testing of NBF-reinforced concrete specimens.

### 2.3. Methods

#### 2.3.1. Workability

The slump cylinder method was employed to assess the workability of the concrete. The concrete was poured into the slump cylinder in three layers, following the guidelines outlined in the Standard Test Methods for Properties of Ordinary Concrete Mixes (GB/T 50080-2016) [38]. Each layer underwent uniform pushing and pounding with the pounding rod from the outer to the central portion in a spiral direction, approximately 25 times per layer. Upon completing the insertion of the top layer, excess concrete on the surface was removed to ensure a flat top surface. Subsequently, the cylinder was smoothly lifted vertically and completely removed within 5–10 s. After removing the cylinder, the slump value was measured using a steel ruler, indicating the height difference between the highest point of the concrete specimen and the height of the cylinder.

#### 2.3.2. Compressive Strength

In accordance with Standard Test Methods of Physical and Mechanical Properties of Concrete (GB50081-2019) [37], the compressive strength of NBF-reinforced concrete was measured after 28 days of curing, with dimensions of 150 mm × 150 mm × 150 mm. The tests were performed on three identical samples at a loading rate of 0.5–0.8 MPa per second using an electro-hydraulic servo universal testing machine (WAW-600C, Chenda Testing Machine Manufacturing Co., Ltd., Jinan, China).

#### 2.3.3. Tensile Strength

The cubic split tensile strength test of NBF-reinforced concrete was conducted after 28 days of curing, with specimen dimensions of 150 mm × 150 mm × 150 mm. The tests were conducted on three identical samples, applying a loading rate ranging from 0.05 to 0.08 MPa/s using a SANS-CMT5205 universal testing machine (MTS Systems (China) Co., Ltd., Beijing, China).

#### 2.3.4. Flexural Strength

As per the specifications for the prismatic flexural strength test of NBF-reinforced concrete, the dimensions were 100 mm × 100 mm × 400 mm. The test was executed using a SANS-CMT5205 universal testing machine. A consistent and uniform load was applied to the specimen at a loading rate ranging from 0.05 to 0.08 MPa/s.

#### 2.3.5. SEM

Given that this paper focuses mainly on analyzing the types and distribution of hydration products on NBF in cement-based materials, SEM was exclusively utilized to observe their morphology for the identification and analysis of hydration products. Specifically, Ca(OH)_2_; appears as regular crystals in flake form, while hydrated calcium silicate manifests as agglomerates or needle-shaped structures, demonstrating distinct morphological differences between them.

The microstructure of NBF-reinforced concrete was analyzed using a COXEM EM-30AX+ scanning electron microscope (COXEM, Daejeon, Republic of Korea). Crushed pieces of NBF-reinforced concrete from the compressive test were carefully selected and prepared as microscopic analysis samples for measuring. To ensure the apparent physical phase morphology of the specimens, the natural section without polishing and grinding was utilized. Before observation, the specimens underwent drying in an electric blast drying oven, and any particles on the surface were cleaned with a brush. Subsequently, they were gold-coated, and SEM images were captured under vacuum conditions.

## 3. Orthogonal Experimental Design

Orthogonal experimental design is an approach used to investigate multi-factor and multi-level experiments, emphasizing representative points in a comprehensive test. The chosen tests are “neat, comparable, and evenly dispersed.” This method is commonly employed for tackling multifactorial issues and offers the benefits of economy, speed, and high efficiency.

An orthogonal experimental design was adopted with the NBF length, NBF content, and water-to-cement ratio as variables. Each factor was tested at three levels using the L_9_(3^3^) orthogonal experimental design table, as shown in Table 4. Nine sets of experiments were conducted with nine standard test blocks for each mixing proportion, resulting in a total of 81 test blocks. The test results were analyzed using range analysis to determine the order of factors affecting the test and the optimal level of each factor, thereby obtaining the optimal ratios. Meanwhile, analysis of variance with repeated tests was used for data processing to analyze whether the effect of each factor on the indices was significant.

## 4. Results and Discussions

### 4.1. Range Analysis of Orthogonal Experiment

Table 5 shows the NBF-reinforced concrete’s 28-day results of compressive strength, splitting tensile strength, flexural strength, and slump value. It is evident that the compressive strength, flexural strength, and splitting tensile strength of NBF-reinforced concrete increased by 11.8%, 15.5%, and 20.3%, respectively, with an increase in the fiber admixture from 0.2% to 0.4% by volume. Conversely, an increase in the water–cement ratio from 0.55 to 0.60 resulted in a decline in the compressive strength of NBF-reinforced concrete by 3.6%, a decrease in flexural strength by 11.3%, and a reduction in splitting tensile strength by 8.9%. The results of the range analysis are outlined in Table 6. Taking the cubic compressive strength as an example, the relationship between the range values *R* of factors A, B, and C decreased in the following order: C (3.97) > A (3.64) > B (3.50). Therefore, the relative importance of factors influencing the compressive strength of NBF-reinforced concrete decreased as follows: water-to-cement ratio > NBF length > NBF content. In addition, the average values *k*_11_, *k*_12_, and *k*_13_ of the three levels of factors A, B, and C were obtained. The level with the highest average value for each factor was determined as the optimal level for that factor. For the compressive strength of NBF-reinforced concrete, the optimal levels of the influencing factors were determined as a fiber length of 10 mm, a fiber content of 0.4v%, and a water-to-cement ratio of 0.55, constituting an optimal level combination denoted as A1B3C1. Similar procedures were followed for the range analysis of other indicators.

**Figure 5:**
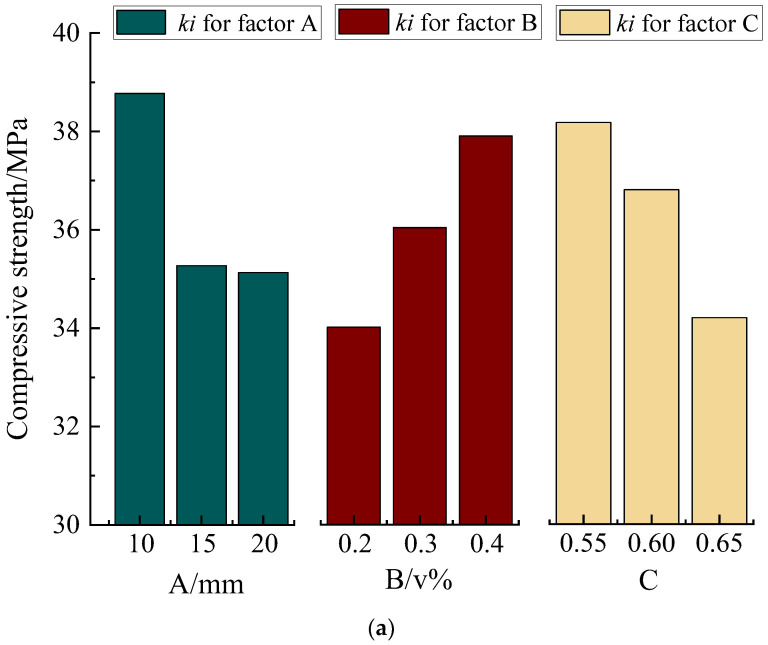

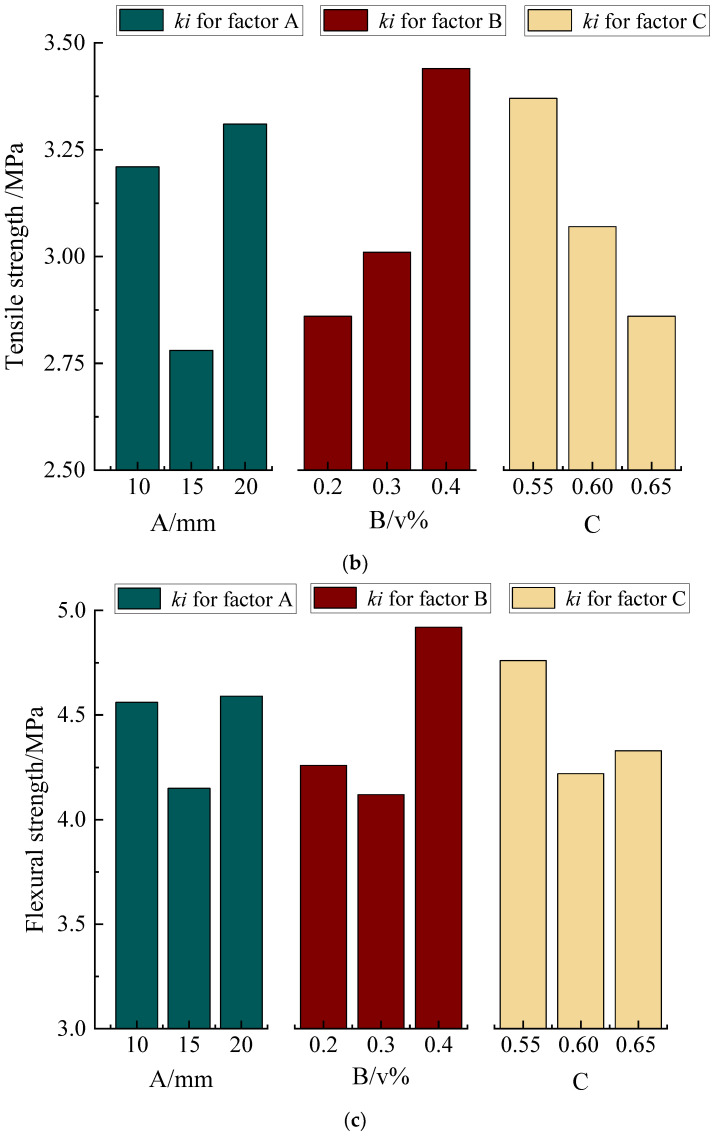

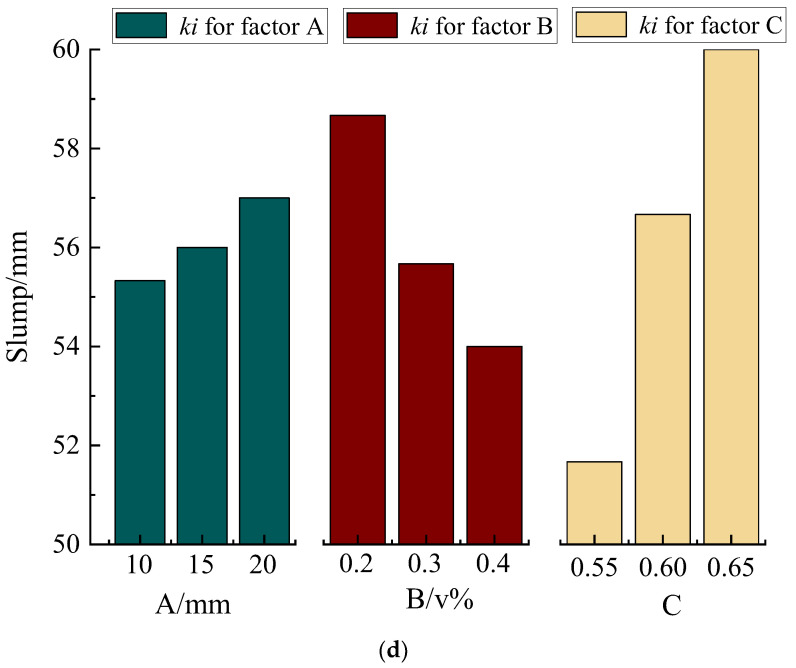
Mean effects of various indicators: (**a**) compressive strength, (**b**) splitting tensile strength, (**c**) flexural strength, and (**d**) slump.

The mean effects are plotted in Figure 5, allowing for the visual observation of the relative importance of factors affecting various indicators. In Figure 5, the maximum value in the bar graph represents the optimal level to choose. A summary is provided in Table 7. For compressive strength and slump, factor C had the largest range *R*, with values of 3.97 and 8.33, respectively. For tensile strength and flexural strength, factor B had the largest range *R*, with values of 3.44 and 0.80, respectively. Thus, we can conclude that the main factors influencing the mechanical properties and workability were NBF content and the water-to-cement ratio, with fiber length having a relatively small effect. Based on these findings, the optimal level combination was determined as A3B3C1, meaning that the optimal properties were obtained with an NBF length of 20 mm, an NBF content of 0.4v%, and a water-to-cement ratio of 0.55. The presence of two entries labeled as A3B3C1 indicates that for split tensile strength and flexural strength, choosing factor A at level 3, factor B at level 3, and factor C at level 1 are all considered optimal choices.

### 4.2. Variance Analysis of Orthogonal Experiment

Range analysis allowed for the determination of the primary and secondary factors along with the optimal level combination. However, it could not distinguish between fluctuations caused by changes in factor levels from those caused by experimental errors. Therefore, analysis of variance with repeated measures was used for data processing to analyze whether the effects of each factor on the indicators were significant. The results are presented in Table 8, where * indicates that the factor had a significant influence, and (*) indicates that the factor had some influence. As indicated in Table 8, the NBF length, NBF content, and water-to-cement ratio exerted significant influences on compressive strength, while fiber content significantly affected flexural strength. Regarding tensile strength, the influence of each factor was not significant, although fiber content exhibited the largest effect among the factors. Additionally, the primary factor impacting slump was the water-to-cement ratio, consistent with the range analysis.

### 4.3. Strength and Failure Forms of NBF-Reinforced Recycled Aggregate Concrete

Using PO42.5 ordinary Portland cement, Grade II fly ash, sand, recycled aggregate, NBFs, and water as raw materials, NBF-recycled aggregate concrete was prepared according to the optimal combination of A3B3C1 obtained from the orthogonal experiment. To investigate the NBF’s influence on the properties of recycled aggregate concrete, two experimental groups were established: one with NBFs and one without. Three specimens with dimensions of 150 mm × 150 mm × 150 mm were prepared, and their compressive strengths were tested after 28 days of standard condition curing.

The recycled aggregate concrete compressive strength with and without NBF were assessed, after curing the test blocks in a standard curing condition for 28 days. The NBF-reinforced recycled aggregate concrete exhibited a compressive strength of 25.2 MPa, whereas the compressive strength was 23.4 MPa without being NBF-reinforced. Consequently, blending NBFs with lengths of 20 mm into the concrete at a content of 0.4v% resulted in an overall increase in compressive strength of 7.3%.

Bamboo fibers contribute to increased compressive strength and play a crucial role in limiting crack propagation in concrete, aligning with the findings of previous studies. Kumarasamy et al. [39] observed that bamboo fiber-reinforced concrete with fiber content at 0.5%, 1%, and 1.5% resulted in increments in compressive strength of 0.9%, 1.38%, 1.73%, and 1.98%, respectively. Similarly, Jayaprakash et al. [40] reported a maximum compressive strength of 26.4 MPa (28 days) with 1.5% bamboo fiber.

**Figure 6:**
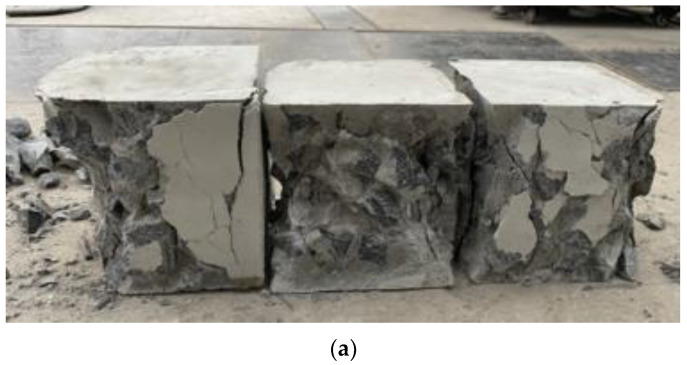

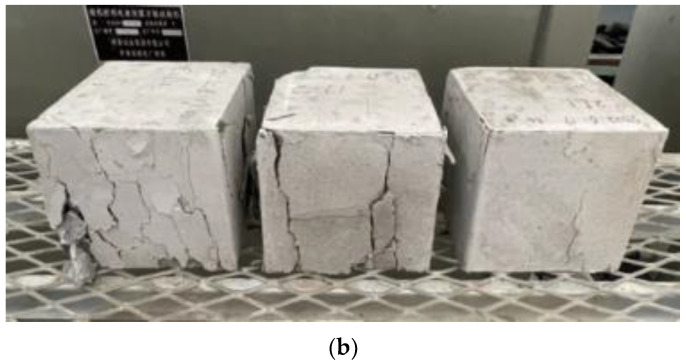
Failure patterns of compressive damage: (**a**) recycled aggregate concrete without NBFs and (**b**) recycled aggregate concrete with NBFs.

Figure 6 shows recycled aggregate concrete failure patterns with and without NBFs. The plain concrete control exhibited a typical inverted quadrangular conical failure pattern, forming two opposing cone-shaped failure surfaces with evident characteristics of brittle failure. The incorporation of NBFs inhibited crack development to a certain extent. The failure pattern was mainly the outward expansion of the entire surface mortar layer, indicating that the fibers tied the concrete together. After failure, the specimens exhibited good overall integrity with a relatively complete form and no local collapse.

Although the improvement may not be as pronounced as steel fiber, the NBF significantly enhances both concrete tensile strength and toughness, playing a crucial role in restraining crack propagation and postponing failure.

### 4.4. Microstructural Analysis

To investigate the mechanism by which NBFs reinforce cement-based materials, the crushed pieces of NBF-reinforced recycled aggregate concrete after compression failure were made into microanalysis specimens.

**Figure 7:**
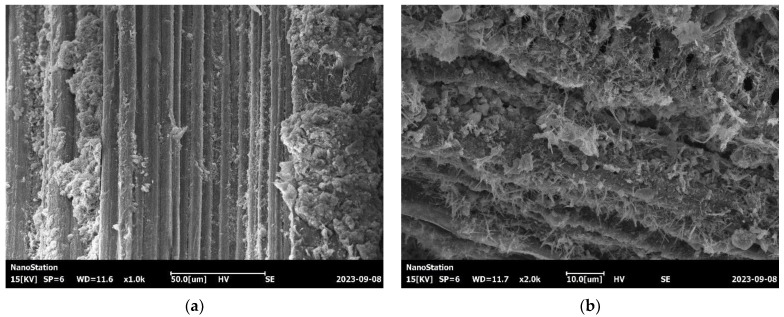
SEM images of NBF-reinforced concrete: (**a**) 1000× magnification and (**b**) 2000× magnification.

The observation results in Figure 7 show that the fiber surface was covered with dense needle-like and granular hydration products that were tightly wrapped around the fibers, resulting in the formation of mechanical interlocking between the NBFs and the cement matrix. When magnified 2000 times, the microstructural features of the fiber–paste interface could be clearly seen. This transition zone exhibited small gaps and tight bonding with almost no flaky Ca(OH)_2_ crystals, which is beneficial for strength because flaky crystals lead to a loose interface. These observations can be attributed primarily to the hydrophilicity and hygroscopicity of the NBFs, which resulted in a lower water-to-cement ratio in the NBF–paste interface, thereby limiting the Ca(OH)_2_ crystals’ formation. The hydration products were mainly hydrated calcium silicate (C–S–H) gel and needle-shaped ettringite; the former connected the two sides of the interface, and the latter were intertwined in a granular form, greatly increasing the structural density and enhancing the bond between the fibers and the cement matrix. As a result, the NBFs had the beneficial effect of tying the concrete structure together.

**Figure 8:**
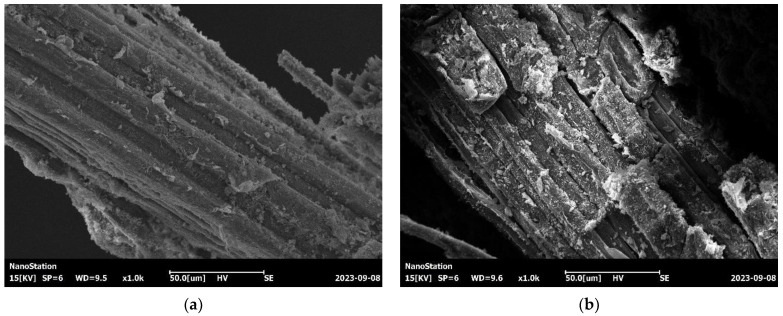
SEM images of NBFs: (**a**) age of 28 d and (**b**) age of one year.

The filamentous NBFs retained the original natural physical bamboo fiber structure. Figure 8 demonstrates the NBF’s microscopic morphology, revealing exposed fiber bundles. Due to the alkaloid treatment, substances such as hemicellulose, lignin, and fructose pectin in the fiber bundles were dissolved, leaving behind highly crystalline cellulose [41]. Figure 8 presents the NBF’s morphology at ages of 28 days and one year. The NBFs in Figure 8b had relatively rough surfaces but still maintained good integrity. This is because the main cause of fiber embrittlement is the migration of Ca(OH)_2_ into the fiber cavities and walls, leading to fiber mineralization [42]. When observing the fiber–paste interface, Ca(OH)_2_ was almost absent. Thus, the alkaloid-treated NBFs underwent a slow embrittlement process in the cement-based materials, allowing them to maintain good performance over time.

During the preparation of the scanning electron microscope specimens of NBF-reinforced concrete aged for 1 year, it was observed that the bond between the fibers and the cementitious material was not particularly robust. This issue may arise from the hydrophilic nature of bamboo fibers, making them prone to wet expansion and dry contraction, which can result in deformation or cracking of the composite material [43]. Minimizing moisture absorption and enhancing volume stability are crucial for facilitating the widespread utilization of NBFs. Remarkably, they exhibit remarkable potential as internal curing materials, absorbing free water at an early stage and supplying the necessary water for the cement hydration reaction and thereby reducing cracking caused by drying shrinkage of the concrete.

## 5. Conclusions

This study developed sustainable and cost-effective concrete incorporating recycled aggregates and natural bamboo fibers. The findings indicate that natural bamboo fiber-reinforced recycled aggregate concrete is sustainable, cost-effective, and enhances the mechanical properties of the construction material. The following conclusions can be inferred:

(1) The optimal mixing parameters obtained from the orthogonal experiment were as follows: a fiber length of 20 mm (considering lengths of 10 mm, 15 mm, and 20 mm), a fiber content of 0.4v% by volume (considering 0.2v%, 0.3v%, and 0.4v%), and a water-to-cement ratio of 0.55 (considering ratios of 0.55, 0.60, and 0.65). The primary factors influencing the mechanical properties and workability were fiber content and the water-to-cement ratio, with the effect of fiber length being relatively minor.

(2) The optimal average compressive strength of NBF-reinforced recycled aggregate concrete is 25.2 MPa, representing a 7.3% increase compared to 23.4 MPa for recycled concrete without NBF.

(3) The fibers played a role in toughening and resisting cracks within the concrete, thereby enhancing its resistance to brittleness. Under compression, the predominant failure pattern of NBF-reinforced concrete involved the outward expansion of the entire surface mortar layer, with the fibers effectively binding the concrete together. The specimens exhibited good overall integrity after failure, maintaining their complete form without localized collapse.

(4) At the NBF-hardened cement–paste interface, minimal flaky Ca(OH)_2_ crystals were observed, while the hydration products primarily comprised needle-shaped ettringite and hydrated C–S–H gel. These products intertwined to enhance structural density and facilitate effective bonding at the NBF-hardened cement–paste interface.

This work exclusively focuses on the types and distribution of hydration products in NBF analysis in cement-based materials using SEM for observing their morphology and identifying and analyzing hydration products. In future research, we plan to integrate SEM, XRD, and FTIR techniques to conduct a more comprehensive and scientifically rigorous analysis of the micro-mechanisms of materials. We will carefully study and address this issue in depth in our follow-up research.

The interfacial debonding of NBF in cementitious materials due to fiber water absorption has not been explored in this paper. Therefore, further research is warranted to evaluate the NBF-reinforced concrete outdoor performance under both ultraviolet rays and humidity variations. Additionally, investigations should be conducted on the mechanism of action of modifiers on the fiber surface and their influence on interfacial bonding to expand the potential application.

## Figures and Tables

**Table 1:** Sieving results of recycled aggregate.

Particle Size (mm)	31.50	26.50	19.00	16.00	9.50	4.75	2.36
Actual individual percent retained (%)	4.9	15.1	42.7	16.8	20.2	0.1	0.1
Actual cumulative percent retained (%)	4.9	20.0	62.7	79.5	99.7	99.8	99.9
Standard cumulative percent retained (%)	0–5	/	15–45	45–70	70–90	90–100	95–100

**Table 2:** Basic properties of recycled aggregate.

Water Absorption (%)	Crushing Index (%)	Apparent Density (kg/m^3^)
5.2	14.6	2520

**Table 3:** C30 concrete mixing proportions.

Water–Cement Ratio	Cement (kg/m^3^)	Fly Ash (kg/m^3^)	Sand (kg/m^3^)	Recycled Aggregate (kg/m^3^)	Water (kg/m^3^)
0.55	290.4	72.6	671.9	1194.5	200.0
0.60	266.6	66.7	682.8	1213.9	200.0
0.65	246.2	61.5	692.0	1230.3	200.0

**Table 4:** Orthogonal experimental factor levels.

Level	Factor		
	A: Fiber Length (mm)	B: Fiber Content (v%)	C: Water-to-Cement Ratio
1	10	0.2	0.55
2	15	0.3	0.60
3	20	0.4	0.65

**Table 5:** Testing results of indicators in the orthogonal experiment.

Test	A (mm)	B (v%)	C	Compressive Strength (MPa)	Tensile Strength (MPa)	Flexural Strength (MPa)	Slump (mm)
1	1 (10)	1 (0.2)	1 (0.55)	38.5	3.12	4.81	57
2	1	2 (0.3)	2 (0.60)	39.6	2.98	4.02	53
3	1	3 (0.4)	3 (0.65)	38.2	3.54	4.84	56
4	2 (15)	1	2	33.4	2.75	3.66	58
5	2	2	3	32.6	2.32	3.84	63
6	2	3	1	39.4	3.28	4.96	47
7	3 (20)	1	3	31.8	2.72	4.30	61
8	3	2	1	36.6	3.72	4.50	51
9	3	3	2	37.0	3.49	4.97	59

**Table 6:** Range analysis of the orthogonal experimental results.

Index		*K*_11_	*K*_12_	*K*_13_	*k*_11_	*k*_12_	*k*_13_	*R*
Compressive strength	A	116.3	105.8	105.4	38.77	35.27	35.13	3.64
	B	104.1	108.8	114.6	34.70	36.27	38.20	3.50
	C	114.5	110.4	102.6	38.17	36.80	34.20	3.97
Tensile strength	A	9.64	8.35	9.93	3.21	2.78	3.31	0.53
	B	8.59	9.02	10.31	2.86	3.01	3.44	0.58
	C	10.12	9.22	8.58	3.37	3.07	2.86	0.51
Flexural strength	A	13.67	12.46	13.77	4.56	4.15	4.59	0.44
	B	12.77	12.36	14.77	4.26	4.12	4.92	0.80
	C	14.27	12.65	12.98	4.76	4.22	4.33	0.54
Slump	A	166	168	171	55.33	56.00	57.00	1.67
	B	176	167	162	58.67	55.67	54.00	4.67
	C	155	170	180	51.67	56.67	60.00	8.33

**Table 7:** Relative importance of factors influencing each indicator.

Index	Relative Importance	Optimal Level Combination
Compressive strength	C > A > B	A1B3C1
Splitting tensile strength	B > A > C	A3B3C1
Flexural strength	B > C > A	A3B3C1
Slump	C > B > A	A3B1C3

**Table 8:** Analysis of variance for NBF-reinforced concrete properties.

Index	Source of Variance	Sum of Squares of Deviation	Degrees of Freedom	Mean Square	*F*	Significance	Critical Value of *F*
Compressive strength	A	25.469	2	12.734	18.515	*	*F*_0.1_(2,2) = 9
	B	18.442	2	9.221	13.407	*	*F*_0.05_(2,2) = 19
	C	24.362	2	12.181	17.711	*	*F*_0.01_(2,2) = 99
	Error	1.376	2	0.688			
Tensile strength	A	0.472	2	0.236	1.904		*F*_0.1_(2,2) = 9
	B	0.534	2	0.267	2.156	(*)	*F*_0.05_(2,2) = 19
	C	0.399	2	0.199	1.611		*F*_0.01_(2,2) = 99
	Error	0.248	2	0.124			
Flexural strength	A	0.354	2	0.177	5.516		*F*_0.1_(2,2) = 9
	B	1.108	2	0.554	17.248	*	*F*_0.05_(2,2) = 19
	C	0.489	2	0.244	7.603	(*)	*F*_0.01_(2,2) = 99
	Error	0.064	2	0.032			
Slump	A	4.222	2	2.111	0.0709		*F*_0.1_(2,2) = 9
	B	33.556	2	16.778	0.563		*F*_0.05_(2,2) = 19
	C	105.556	2	52.778	1.772	(*)	*F*_0.01_(2,2) = 99
	Error	59.556	2	29.778			

## Data Availability

Data will be made available on request.
